# Alteration of movement patterns in low back pain assessed by Statistical Parametric Mapping

**DOI:** 10.1016/j.jbiomech.2019.109597

**Published:** 2020-02-13

**Authors:** Enrica Papi, Anthony M.J. Bull, Alison H. McGregor

**Affiliations:** a Department of Surgery and Cancer, Imperial College London, London, UK; b Department of Bioengineering, Imperial College London, London, UK

**Keywords:** Kinematics, Time series analysis, Range of motion, 3D motion analysis

## Abstract

Changes in movement pattern in low back pain (LBP) groups have been analysed by reporting predefined discrete variables. However, this approach does not consider the full kinematic data waveform and its dynamic information, potentially exposing the analysis to bias. Statistical Parametric Mapping (SPM) has been introduced and applied to 1 dimensional (D) kinematic variables allowing the assessment of data over time. The aims of this study were to assess differences in 3D kinematics patterns in people with and without LBP during functional tasks by using SPM and to investigate if SPM analysis was consistent with standard 3D range of motion (RoM) assessments. 3D joints kinematics of the spine and lower limbs were compared between 20 healthy controls and 20 participants with non-specific LBP during walking, sit-to-stand and lifting. SPM analysis showed significant differences in the 3Dkinematics of the lower thoracic segment, upper and lower lumbar segment and knee joint during walking and lifting mostly observed at the beginning and/or towards the end of the tasks. ROMs differed between groups in the lower thoracic segment (walking/sit-to-stand), upper and lower lumbar segments (walking/sit-to-stand/lifting), hip and knee (sit-to-stand/lifting). Based on these results, the two approaches can yield different data interpretations. SPM analysis allows the identification of differences in movement that occur over time. This adds value to LBP movement analysis as it allows an understanding of the LBP strategies adopted during motion that may not be conveyed by simple discrete parameters such as ROMs.

## 1 Introduction

Low back pain (LBP) is the leading cause of disability worldwide (Hartvigsen et al., 2018). Although its aetiology in the majority of cases is unclear (van Krismer and Tulder (2007)), there is a possible causative role between posture and movement and LBP development (Van Dillen et al., 2003).

Movement in the LBP population has been extensively reported using movement analysis studies. Frequently discrete parameters have been compared, such as range of motion (ROM) and values at particular instants of the analysed task cycles (Papi et al., 2018). However, using predetermined parameters to verify undirected test hypotheses exposes the data to biases: ‘regional focus bias’ and ‘inter-component covariation bias’ (Pataky et al., 2013, Pataky et al., 2016a, Pataky, 2016b). By concentrating the analysis to 0-Dimensional (0D) scalar parameters the entire measurement domain is not taken into account, and therefore differences during other instances of the task or along the time dimension may be missed (regional focus bias) (Pataky et al., 2013, Pataky et al., 2016a, Pataky, 2016b). Moreover, the 3D components which describe joints movement may not be independent, thus analysing these separately as scalar parameters ignores signal covariance, another source of bias (inter-component covariation bias) (Pataky et al., 2013). Extending the analysis to the entire time series of a given movement task could enhance the understanding of the strategies adopted and how different population groups (e.g. healthy vs LBP groups) use the different degrees of freedom available to achieve the same functional task, which cannot be inferred from discrete values. The examination of the entire movement is akin to clinical assessment where a clinician observes the patient performing a task. However, 0D statistical approaches (e.g. *t*-test) cannot be applied at each time point of a kinematic time series (1D vector) when the study hypothesis pertains to 1D vectors rather than to a particular instant of the task analysed (Pataky et al., 2015). The 0D probability distribution is different from an n-Dimensional distribution and therefore applying 0D statistical method to 1D data (e.g.: time series) results in an increased number of false positives (Pataky et al., 2015).

To solve these issues, Statistical Parametric Mapping (SPM) has been proposed for hypothesis testing of 1D data (Pataky et al., 2013). SPM was originally used in brain research (Friston et al., 2007), and has now also been applied to the analysis of biomechanical datasets (Li et al., 2016, Goudriaan et al., 2018, Malfait et al., 2016, Robinson et al., 2014, Vanrenterghem et al., 2012). SPM exploits Random Field Theory (Adler and Taylor, 2007) to determine statistical inference over 1D continuous vector trajectories and its application has been previously validated (Pataky et al., 2016a; Pataky, 2016b). SPM allows presentation of statistical outputs in the original 1D domain (either time or space), providing an understanding of temporal/spatial regions where significant differences may occur. Therefore, implementation of this technique into LBP movement research allows analysis of the whole kinematic waveforms, thus retaining the full movement interval. Previous studies that report kinematic waveform comparisons between control and LBP groups do not apply any statistical approach to assess differences between the curves on a point-by-point basis but rather offer a qualitative assessment of the curves (Christe et al., 2016, Christe et al., 2017, Crosbie et al., 2013b, Müller et al., 2015). These are then used to extrapolate kinematics parameters or perform coordination analyses of segments/joints movement between controls and LBP participants which are then compared statistically.

Therefore, the aims of this study were to use SPM to assess differences in 3D joint kinematics patterns in people with and without LBP during a series of functional tasks on a point-by-point basis and to qualitatively investigate if ROM statistical analysis was consistent with SPM analysis outputs. The null hypothesis tested is that there are no differences between the two groups assessed. In light of recent suggestions on LBP movement studies (Christe et al., 2016, Christe et al., 2017, Gombatto et al., 2015, Gombatto et al., 2017, Papi et al., 2019, Papi et al., 2018), the analysis was performed using a multi-segmental model of the lumbar and thoracic spine segments, with consideration also given to the lower limbs.

## 2 Material and methods

### 2.1 Participants

Twenty healthy controls (age: 28 ± 7.6 years, body mass: 66.2 ± 12 kg, height: 1.72 ± 0.11 m, 10 female) and 20 patients with non-specific chronic LBP (age: 41 ± 10.7 years, body mass: 74.1 ± 19.5 kg, height: 1.68 ± 10.7 m, 4 female, pain duration range: 10 months – 8 years) were enrolled in this study. Non-specific LBP was defined as pain in the lower back region for which it was not possible to identify a specific cause (e.g. prolapsed disc, sciatica, tumour, spinal stenosis). Participants were excluded if they had neurological diseases, severe musculoskeletal deformities in the lower limbs or spine, spinal fractures, and prior back surgery. Participants used yoga (8), pilates (6), physiotherapy (10) and osteopathy (9) as form of care, with 10 of them using more than one management approach. This study was approved by the North-West Preston Research Ethics Committee and all participants provided written informed consent prior to participation.

### 2.2 Data collection

A 3D motion capture system operating at 100 Hz was used to acquire spine and lower limbs kinematic data (Vicon, Oxford Metrics, Oxford, UK). The marker set used to track the spine and the pelvis has been previously described (Papi et al., 2019). In brief, triads of markers on plastic strips were attached on T1, T6, T7, T12, L1, L3 and L5 and a three-marker rigid cluster was placed over the sacrum. The lower limb marker set comprised four rigid clusters (each cluster with four markers) attached to the distal parts of the left and right thigh and shank segments. All markers were spherical with 14 mm diameter and were positioned by the same researcher for every participant using hypoallergenic double-sided tape. A reference standing posture was collected with participants standing in their natural posture at the beginning of the recording session. Through a static calibration, individual markers on the left and right anterior and posterior iliac spine (ASIS; PSIS), left and right knee epicondyles and ankle malleoli were referenced to the pelvis, thigh and shank clusters respectively and then removed. Participants were then asked to walk at a self-selected pace, perform a sit-to-stand (STS) movement, and lift a 5 kg box. Standardised instructions were used for all participants (Supplementary Material) and each task was repeated 3 times. The tasks were selected as they are daily activities frequently reported as painful for those with LBP and differences have been noted between LBP and healthy groups during such tasks (Christe et al., 2016, Christe et al., 2017, Gombatto et al., 2017, Papi et al., 2018).

Participants with LBP also completed the Oswestry Disability Index (ODI) to quantify disability resulting from their back pain (Fairbank et al., 2000) and the pain during the day of the assessment was evaluated with a visual analogue scale from 0 (no pain) to 10 (extreme pain).

### 2.3 Data processing and analysis

Nexus software (Vicon, Oxford Metrics, Oxford, UK) was used for data pre-processing which included reconstruction, gap filling and filtering of marker trajectories. A Woltring’s general cross-validatory quintic smoothing spline with a predicted mean-squared error of 15 mm was used to filter marker trajectories (Woltring, 1978). Data post-processing was then performed using custom-built Matlab routines (MathWorks, Inc., Natick, USA). Spinal kinematics was calculated based on a previously described multi-segmental model which comprises upper (T1-T6) and lower (T7-T12) thoracic spine segments and upper (L1-L3) and lower (L3-L5) lumbar spine segments (Papi et al., 2019). Pelvis, thigh, shank and foot anatomical reference frames were defined in agreement with standard recommendations (Wu et al., 2002). The hip joint centre was calculated based on Harrington’s regression equations (Harrington et al., 2007) and the knee and ankle joint centres were defined as the midpoint between the medial and lateral epicondyles and the medial and lateral malleoli respectively. The three-cylinder open chain joint coordinate system was used to calculate 3D joint angles of the upper and lower thoracic spine segments, upper and lower lumbar spine segments, pelvis segment, and hip, knee and ankle joints (Grood and Suntay, 1983).

All kinematic data were time normalised to a full task cycle consisting of 100 data points. Gait cycles were defined using the horizontal heel displacement method (Banks et al., 2015) whilst the STS and lifting tasks were defined based on PSIS and T1 markers displacements and velocities as detailed in Papi et al. (2019). The lifting task was split into lowering and picking phases. The average from the 3 repetitions performed for each task of each participant was calculated for subsequent analysis. The left and right sides data were averaged as similar patterns were observed.

The ROMs in all 3 planes for each spine segment and lower limb joints analysed were calculated as the difference between the maximum and minimum angles for the 3 trials of each task performed and then averaged for subsequent comparisons between groups.

### 2.4 Statistical analysis

SPM (Friston et al., 2007) was used to statistically compare the whole kinematic time-series between healthy and LBP groups. A custom Matlab code (MathWorks, Inc., Natick, USA) was used to conduct SPM analyses implementing functions from the open-source spm1d package (www.spm1d.org). Prior to any inferential procedure, data normality was assessed with the built-in function ‘spm1d.stats.normality.ttest’. Depending on data normality, a parametric or non-parametric Hotelling’s T^2^ test was used to compare the 3D (3-component: sagittal, frontal, transverse planes angles) time varying joint kinematics vectors of the spine and pelvis segments and lower limb joints (α = 0.05). An SPM Hotelling’s T^2^ test is the vector-field equivalent to the two-sample *t*-test (Cao and Worsley, 1999, Pataky et al., 2013). The scalar output statistic, SPM{T^2^} or SnPM{T^2^} (non-parametric version), was calculated separately at each individual time point. To test the null hypothesis that there are no differences between groups the critical threshold at which only α % (5%) of smooth random curves would be expected to traverse was calculated. This approach is based on Random Field Theory (Adler and Taylor, 2007) and has been validated for 1D data (Pataky et al., 2016, Pataky, 2016). If significant differences were observed, the 3D time varying joint kinematic vectors were separated into their vector components (e.g. sagittal, frontal, transverse planes angles) and post-hoc analyses performed. Specifically, a parametric or non-parametric two-tailed, two-sample *t*-test (SPM{t} or SnPM{t}) was conducted on sagittal, frontal, transverse planes angles separately. Statistical significance occurs when the SPM curves (SPM{T^2^} or SnPM{T^2^}; SPM{t} or SnPM{t}) cross the critical threshold at any time node. Due to the inter-dependence of neighbouring points, multiple adjacent points of the SPM curves often exceed the critical threshold, these are referred to as “supra-threshold clusters”. Supra-threshold clusters along the SPM curve were identified and the associated p-values were calculated using Random Field Theory. For all non-parametric SPM tests the number of iterations was set at 10,000 (Pataky et al., 2015).

Corrections for multiple tests were not performed across variables for either the ROM analyses or the SPM analyses; this was done deliberately, to maximize power and thus maximize both techniques' chances of discovering true effects.

Participants’ demographics (age, body mass, height) and ROMs were compared by means of independent t-tests or Mann-Whitney U tests depending on data normality (α = 0.05). Data normality was verified using Q-Q plots and the Shapiro-Wilk test.

## 3 Results

ODI questionnaire scores revealed that 5 participants with LBP had moderate disability (21% ≤ ODI ≤ 40%) and 15 minimal disability (ODI ≤ 20%). There were no significant differences between groups in height (p = 0.29) and body mass (p = 0.20), but the LBP group was significantly older than the controls (p < 0.05). LBP participants did not report acute pain at the time of assessment with average pain reported to be 4 ± 2 on a visual analogue scale from 0 (no pain) to 10 (most severe pain).

**Fig. 1:**
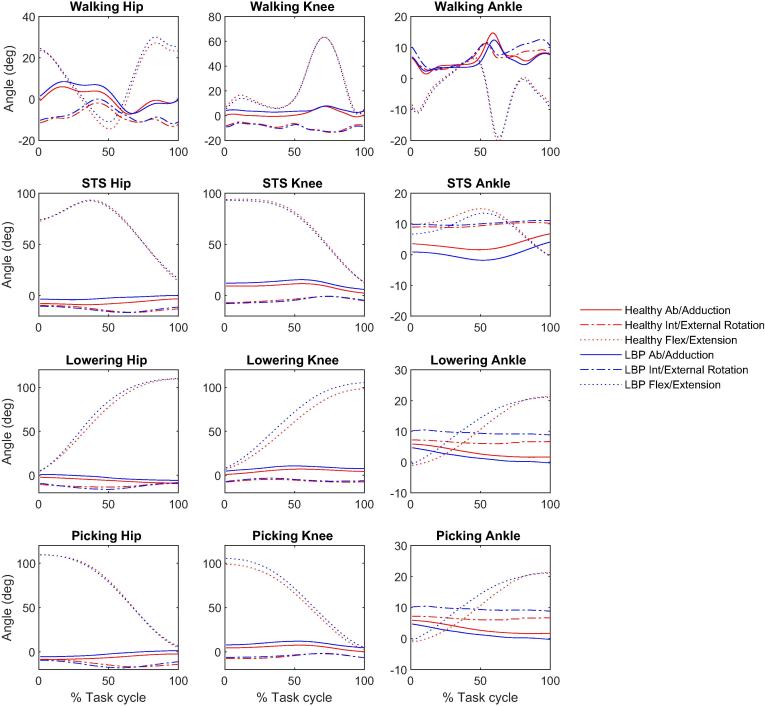
Mean 3D lower limbs joint angles during walking, STS, lifting (lowering and picking phases) for healthy (red lines) and LBP participants (blue lines). (For interpretation of the references to colour in this figure legend, the reader is referred to the web version of this article.)

**Fig. 2:**
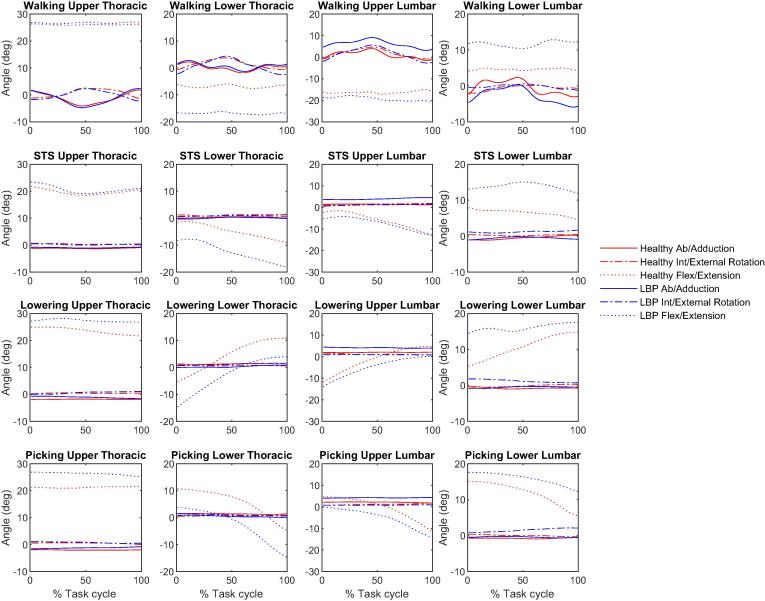
Mean 3D spine segments angles during walking, STS, lifting (lowering and picking phases) for healthy (red lines) and LBP participants (blue lines). (For interpretation of the references to colour in this figure legend, the reader is referred to the web version of this article.)

**Fig. 3:**
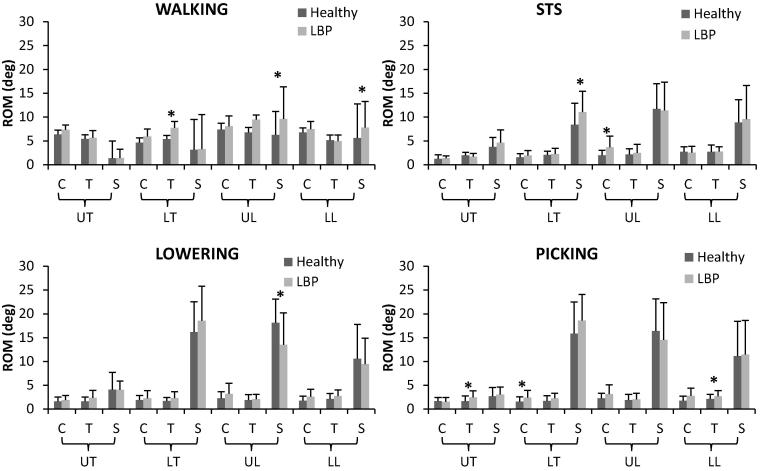
Mean and SD of ROM values in the three anatomical planes (C: coronal, T: transverse. S: sagittal plane) for the spine segments (UT: upper thoracic, LT: lower thoracic, UL: upper lumbar, LL: lower lumbar) for healthy (dark grey bars) and LBP participants (light grey bars). * indicates significant differences.

**Fig. 4:**
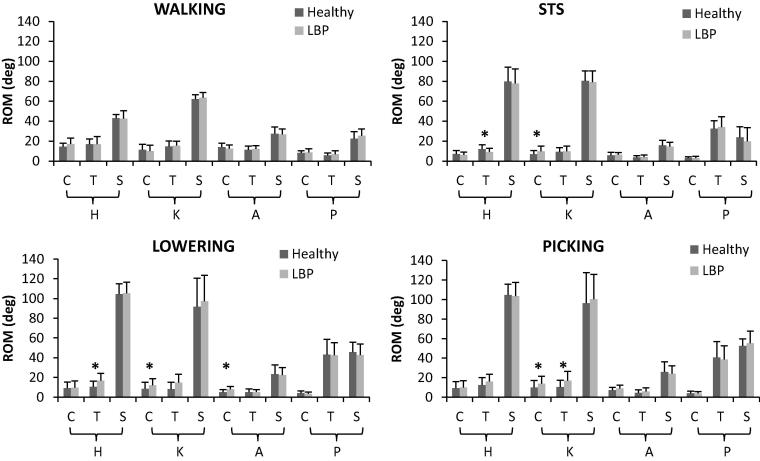
Mean and SD of ROM values in the three anatomical planes (C: coronal, T: transverse. S: sagittal plane) for hip (H), knee (K) and ankle (A) joints and pelvis segment (P) for healthy (dark grey bars) and LBP participants (light grey bars). * indicates significant differences.

Kinematics outputs during the tasks are shown in Fig. 1, Fig. 2 and ROMs are shown in bar charts in Fig. 3, Fig. 4.

### 3.1 Walking

T-tests on ROM values reached significance for the lower thoracic axial rotation (p = 0.03), and upper (p = 0.04) and lower lumbar flexion (p = 0.01), all of which were higher in LBP participants with mean differences of 2.4°, 3.4° and 2.2°, respectively (Fig. 3).

**Fig. 5:**
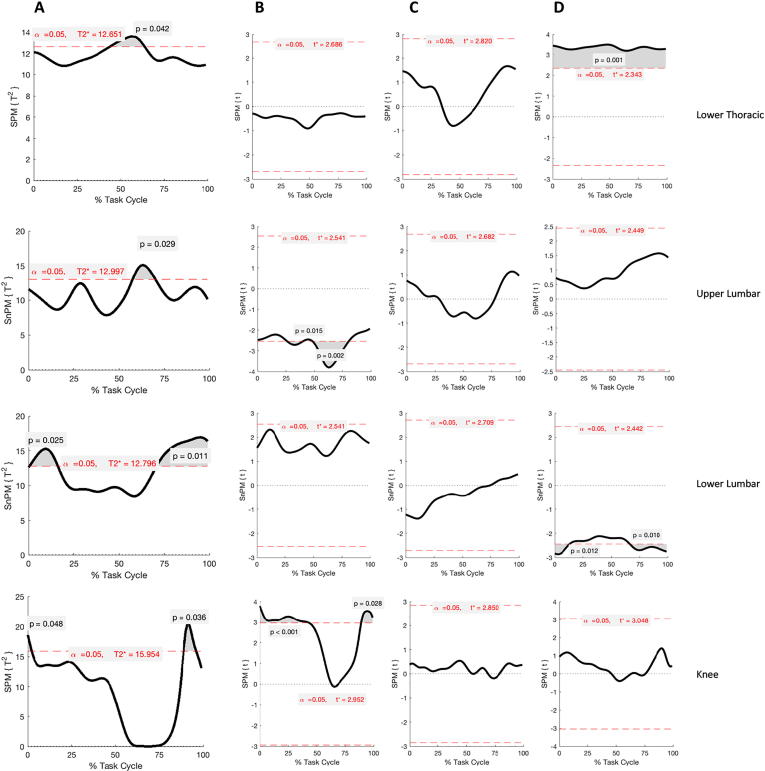
Results of SPM Hotelling T^2^ test parametric and non-parametric (SPM{T^2^} or SnPM{T^2^}, Panel A) and post-hoc analysis (SPM{t} or SnPM{t}, Panel B-D) during walking. Each row refers to a different body segment or joint where significant differences were found. Supra-thresholds clusters indicating significance difference between healthy and LBP participants are shown in grey and the critical threshold as red dashed line. Panels B, C and D show differences in the coronal, transverse and sagittal plane angle respectively from the post-hoc analysis. (For interpretation of the references to colour in this figure legend, the reader is referred to the web version of this article.)

Significant SPM outputs for walking are reported in Fig. 5.

The SPM analysis revealed significant differences in the lower lumbar segment at the beginning (0–16%, p = 0.025) and towards the end (71–100%, p = 0.011) of the gait cycle. Post-hoc analyses suggested that these differences were due to an increase in lower lumbar flexion in the LBP group: two supra-threshold clusters were found in the SnPM{t} curve (0–14%, p = 0.012; 66–100%, p = 0.010).

The upper lumbar segment showed also significant differences between groups in the 58–70% interval (p = 0.029). This was due to differences in lateral bending between the groups where the critical threshold was twice exceeded (26–40%, p = 0.015; 48–81%, p = 0.002).

A supra-threshold cluster (44–64%, p = 0.042) was also found for the 3D kinematic component of the lower thoracic segment. Post-hoc analyses revealed that the sagittal plane angles were dissimilar throughout the entire gait cycle (p = 0.001) with the LBP group exhibiting greater extension of the lower thoracic spine.

The knee joint also exhibited significant differences just after heel strike (0–2%, p = 0.048) and during the swing phase (87–96%, p = 0.036) due to a more adducted knee in the LBP group as shown in post-hoc t-tests (0–43%, p < 0.001; 89–100%, p = 0.028).

### 3.2 Sit-to-Stand

No significant differences were found by SPM analyses during STS. Nonetheless, significant differences were found for lower thoracic flexion ROM (p = 0.03), upper lumbar lateral flexion ROM (p = 0.007), hip rotation ROM (p = 0.02), and knee adduction ROM (p = 0.01) (Fig. 3, Fig. 4). Mean differences were 2.6°, 1.7°, −2.9° and 3.1°, respectively, with positive values indicating bigger ROM for the LBP group.

### 3.3 Lifting

**Fig. 6:**
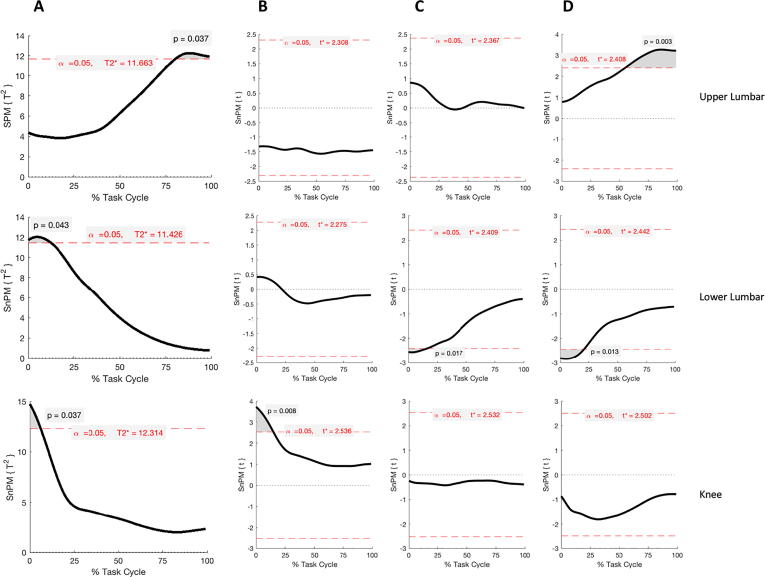
Results of SPM Hotelling T^2^ test parametric and non-parametric (SPM{T^2^} or SnPM{T^2^}, Panel A) and post-hoc analysis (SPM{t} or SnPM{t}, Panel B-D) during the lowering phase of lifting. Each row refers to a different body segment or joint where significant differences were found. Supra-thresholds clusters indicating significance difference between healthy and LBP participants are shown in grey and the critical threshold as red dashed line. Panels B, C and D show differences in the coronal, transverse and sagittal plane angle respectively from the post-hoc analysis. (For interpretation of the references to colour in this figure legend, the reader is referred to the web version of this article.)

**Fig. 7:**
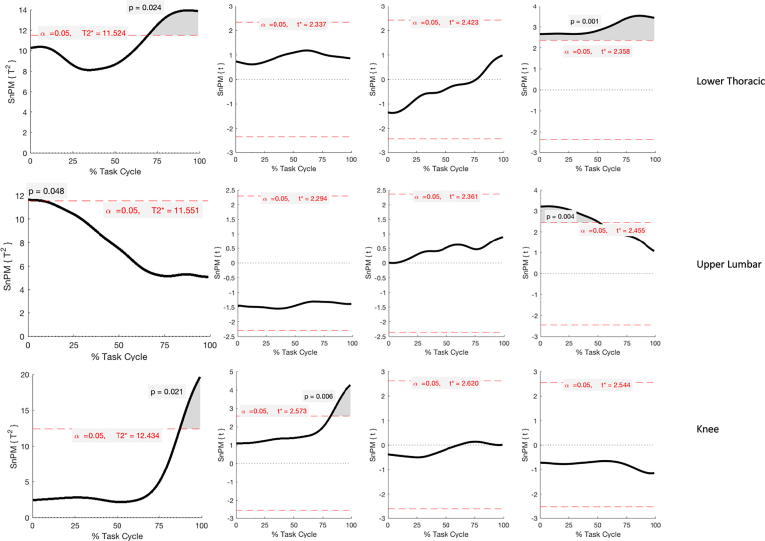
Results of SPM Hotelling T^2^ test parametric and non-parametric (SPM{T^2^} or SnPM{T^2^}, Panel A) and post-hoc analysis (SPM{t} or SnPM{t}, Panel B-D) during the picking phase of lifting. Each row refers to a different body segment or joint where significant differences were found. Supra-thresholds clusters indicating significance difference between healthy and LBP participants are shown in grey and the critical threshold as red dashed line. Panels B, C and D show differences in the coronal, transverse and sagittal plane angle respectively from the post-hoc analysis. (For interpretation of the references to colour in this figure legend, the reader is referred to the web version of this article.)

Significant SPM outputs for the lifting task are reported in Fig. 6, Fig. 7.

During the lowering phase of the lifting task, for the lower lumbar segment one supra-threshold cluster was found to exceed the critical value of t = 11.426 (0–13%, p = 0.043). Post-hoc t-tests revealed differences in both the transverse (0–16%, p = 0.017) and sagittal planes angles (0–21%, p = 0.013).

The upper lumbar segment showed differences towards the end of the lowering phase (80–100%, p = 0.037) due to increased extension in the LBP group (55–100%, p = 0.003).

The knee kinematics also showed differences at the beginning of the lowering cycle (0–6%, p = 0.037) due to a significant increase in knee adduction in the LBP group (0–15%, p = 0.008).

ROMs statistical analysis showed a greater hip rotation (p = 0.07), knee ab/adduction (p = 0.01) and rotation (p = 0.005), and ankle ab/adduction (p = 0.003) in the LBP group compared to the control group with mean differences of 6.2°, 3.7°, 6.5° and 2.9°, respectively. LBP participants, however, had a significant lower upper lumbar flexion (p = 0.036) when compared to controls by −4.7°.

During the picking phase, significant differences were found in the upper lumbar segment (0–9%, p = 0.048) due to reduced flexion in the LBP group (0–51%, p = 0.004).

The lower thoracic kinematic was significantly different between the two groups as demonstrated by one supra-threshold cluster towards the end of the picking cycle (70–100%, p = 0.024). Post-hoc analysis revealed differences throughout the entire picking phase in sagittal plane angles: LBP group had a reduced lower thoracic flexion (0–100%, p = 0.001).

Knee kinematics were significantly different during the picking phase (87–100%, p = 0.021) caused by an increased adduction in the LBP group (82–100%, p = 0.006).

ROM values that reached significance during the picking phase were upper thoracic axial rotation (p = 0.023), lower thoracic lateral flexion (p = 0.03), lower lumbar axial rotation (p = 0.016) and knee ab/adduction (p = 0.03) and rotation (p = 0.06). These were higher in the LBP group by 3.9°, 6.7°, 0.8°, 0.9° and 1.0°, respectively, in comparison to controls.

Differences and similarities between the two analysis methods are summarised in Table 1.

**Table 1:** Summaries of significant differences found with the SPM Analysis and ROM data analysis.

**Significant differences**		
Joints/segments	SPM analysis	ROMs analysis
***Walking***		
Lower thoracic segment	✓	✓
Upper lumbar segment	✓	✓
Lower lumbar segment	✓	✓
Knee	✓	
***STS***		
Lower thoracic segment		✓
Upper lumbar segment		✓
Hip		✓
Knee		✓
***Lowering***		
Upper lumbar segment	✓	✓
Lower lumbar segment	✓	
Hip		✓
Knee	✓	✓
Ankle		✓
***Picking***		
Upper thoracic segment		✓
Lower thoracic segment	✓	✓
Upper lumbar segment	✓	
Lower lumbar segment		✓
Knee	✓	✓

## 4 Discussion

Analysis of LBP movement patterns has been until now focused on statistical analysis of discrete parameters calculated from 1D joint kinematic and/or kinetic vectors. The current study is the first to apply SPM analysis to assess the spine and lower limbs kinematic waveforms of participants with and without LBP during functional tasks. Moreover, it also compares 1D SPM outputs to the statistical analysis of 0D ROM values. The advantage of using SPM is that it allows analysis of the complexity of movement as an entirety.

SPM analysis showed differences during walking and lifting but not during STS. This could be a reflection of the specific instructions that were given to subjects that may have reduced the variability in the movement patterns across participants and groups. Differences in STS ROMs were observed as also reported by Christe et al. (2016) for the spine segments.

The time during which differences occurred could be identified through SPM analysis adding to the LBP literature and our understanding of the strategies adopted by LBP participants. It was observed that most of the differences occurred at the beginning and/or towards the end of the task. This indicates that the initiation and termination of movement is altered in LBP participants showing a different control of their spine and lower limb joints. Thus, whilst LBP subjects can achieve the task, the movement patterns are altered. Although previous literature provides some temporal analysis of peaks occurrence in control and LBP participants during functional tasks (Christe et al., 2016, Christe et al., 2017), SPM extends the analysis to the entire task cycle providing information that may be disguised if the analysis is only performed in relation to a single time point or an arbitrary time window.

Discrete t-tests on ROMs identified differences between the two groups in all 3 tasks performed that were independent of the different time stages of the task. This demonstrates that the two approaches can yield different data interpretations. By concentrating the analysis on ROM values, a task effect was observed between the two groups: 3 significant differences during walking, 4 during STS, and 7 during lifting. This aligns with previous literature based on 0D analyses suggesting that the analysis of demanding activities enhances differences between healthy and LBP groups (Papi et al., 2018, Gombatto et al., 2015). However, this task effect is lost with SPM analysis which was unable to identify any differences in STS, but found 4 differences during walking and 5 during lifting. However, some agreements between SPM and discrete t-tests could also be observed in lower lumbar flexion during walking, knee ab/adduction during both phases of the lifting task and for upper lumbar flexion during the lowering cycle. Discrepancies between the two approaches adopted have been reported in other studies comparing 0D and 1D analyses of kinematic data (Pataky et al., 2013, Pincheira et al., 2019, Sole et al., 2017). Peaks and troughs, used in ROM calculation, may occur at different time points among individuals (e.g. are not aligned) and these differences may be cancelled out when considering the whole-time domain of the kinematic vector (Sole et al., 2017) explaining why some of the differences observed in discrete *t*-test analysis were not reflected in SPM. This highlights the importance of always associating ROM analysis with a temporal analysis of when peaks and troughs occur such as also performed in recent studies (Christe et al., 2016, Christe et al., 2017), otherwise important information would be missed. Moreover, 0D thresholds are lower than the 1D threshold, and whilst more sensitive to small changes they are also responsible for an increased number of false positives (Pataky et al., 2015). Finally, since we did not design our study to investigate predefined discrete variables it would be incorrect to apply 0D statistical analysis to our data but rather retain all the information and conduct statistical analysis over the whole kinematic time domain as per SPM analysis. In this way, we do not incur the risk of overlooking important information related to LBP pathomechanics by selecting discrete variables to address a study hypothesis that refers to the whole-body kinematics.

Comparison with previous literature is somewhat difficult as the majority of studies do not report kinematic data of regions other than the lumbar spine, frontal and transverse plane rotations, and the entire joint/segment kinematic waveform (Papi et al., 2018). Nevertheless, in agreement with prior literature, kinematic differences were observed at the lower thoracic level during walking and lifting (Christe et al., 2017, Crosbie et al., 2013a, Crosbie et al., 2013b). Our study expands prior results by showing a persistent significant reduced lower thoracic flexion throughout the tasks cycles. Coronal and transverse plane rotations were not significantly different over time but showed ROMs higher in LBP as in Crosbie et al., 2013a, Crosbie et al., 2013b). The upper lumbar segment was also less flexed in LBP but this was significant only during lifting in the second and first half of the lowering and picking tasks respectively. Conversely, similar movement restrictions were not observed in the lower lumbar region as reported elsewhere for ROM values for walking and lifting (Christe et al., 2017, Gombatto et al., 2017). The increased lower lumbar flexion, observed in our LBP participants, could explain the reduced lower thoracic and upper lumbar flexion. During walking the lower lumbar segment was significantly more flexed at the beginning and towards the end of the gait cycle, and at the beginning of the lowering phase of the lifting task in the LBP group. These results are novel as no previous study provides statistical analysis of the lower lumbar spine kinematics time series making direct comparison challenging. Results on lumbar segments kinematics are contradictory in the literature with some reporting movement restrictions whilst others not (Papi et al., 2018). Moreover, previous studies lack a contextualisation of what is happening across the body to fully explain their findings. Our results also showed a trend in the LBP group to maintain the hips in a more flexed position. This indicates how the LBP group maintains a rigid upper lumbar and thoracic spine, as a protective mechanism, whilst locking the hip flexors contributing to a more flexed lumbar spine. Hip flexor dominance is also seen clinically in LBP therefore explaining the increased lower lumbar flexion seen in our LBP group. This substantiates previous findings alluding to compensatory hip movement pattern in LBP groups (Crosbie et al., 2013a, Gombatto et al., 2017). Moreover, our LBP participants did not have acute pain at the time of testing and this could further explain the lack of movement restriction at the lower lumbar level.

The LBP group also displayed a significant increase in knee adduction during walking and lifting as also reported by Gombatto et al. (2017). Anecdotally, clinicians report that LBP groups rely more on hip flexors and hamstrings than gluteus and lower abdominal muscles, and weaker gluteus muscles could contribute to the greater knee adduction observed (Król et al., 2017). Increased knee adduction may result in a greater load on the knee and could be one of the reasons for the increased risk of lower limb injuries in LBP (Leung et al., 2015). Rehabilitation programmes should therefore consider the whole-body and not just the lumbar spine as compensatory movements in other body regions could lead to additional injuries. Moreover, movement functions in all anatomical planes should also be considered as our findings suggest that movement alterations occur also in the frontal and transverse planes.

There are limitations in this study; the use of skin markers may have affected kinematics outputs due to soft tissue artefacts which were not accounted for. However, both groups were exposed to both the instrumentation and soft tissue artefacts therefore it is not expected that the differences found are due to those errors. Nevertheless, experimental errors could have occurred and concealed further differences between the two groups. Participants with LBP showed low to moderate disabilities and therefore more differences could be expected in a more severe group. Participants did not receive MRIs as part of this study to confirm the absence of a specific cause for their LBP. Finally, gender differences were not accounted for in the analysis and the age gap between the two groups could theoretically have also altered kinematics outputs. However, as speed is affected by age and could affect joint kinematics (Schmid et al., 2017), and both groups exhibited similar speeds during the tasks performed (p-values range: 0.13–0.74), the age gap between the groups may not be important in this study.

In conclusion, SPM analysis yielded significant differences in the 3D kinematic waveforms between participants with and without LBP. Significant differences were observed in the lower thoracic, upper and lower lumbar spine segments but also in the knee, especially during the initiation and termination phases of the walking and lifting tasks. These findings add value to the current LBP literature as they allow an understanding of when the differences in movement patterns occurred which may not be conveyed by simple discrete parameters.

## Declaration of Competing Interest

The authors declare that they have no known competing financial interests or personal relationships that could have appeared to influence the work reported in this paper.
