# Supplementary material for: Alteration of movement patterns in low back pain assessed by Statistical Parametric Mapping
Source: J Biomech. 2020 Feb 13;100:109597. doi: 10.1016/j.jbiomech.2019.109597 (PMC7001037; doi:10.1016/j.jbiomech.2019.109597)
Supplement: Supplementary data 1 [file mmc1.docx]

| **Tasks** | **Procedure** | **Instruction** |
| --- | --- | --- |
| Walking at a comfortable speed | Subjects will walk along a 6m walkway at a comfortable speed. They will be unaware of the two force plates; ask them to walk as naturally as possible. Allow them to practice as many times as necessary.  3 x Walking with left & right foot contact with force plates. | “When you will hear, ‘GO’, please walk to the end of the walkway as you would usually walk at your preferred speed with your arms relaxed by your side and looking ahead.” |
| Lifting task | Place a cardboard box (5 kg) on the floor in front of where the subject is standing, close to his/her toes. Subject will be standing with each foot on a force plate.  3 x lifting task | “When you will hear, ‘GO’, please lift the box in front of you to the level of your waist” |
| Sit to Stand | Place the height adjustable backless chair adjacent to two force plates. Subjects will be seated with each foot positioned on one of the two force plates hip width apart. Adjust the height of the chair to allow each subject’s thigh to be horizontal and their shank vertical.  3 x sit-to-stand task | “When you will hear ‘GO’, please stand up with your arm crossed over your chest and remain standing. You will perform this movement in what you consider a normal way, at your own preferred speed” |
