# Supplementary material for: Alteration of movement patterns in low back pain assessed by Statistical Parametric Mapping
Source: J Biomech. 2020 Feb 13;100:109597. doi: 10.1016/j.jbiomech.2019.109597 (PMC7001037; doi:10.1016/j.jbiomech.2019.109597)
Supplement: Supplementary data 2 [file mmc2.docx]

The graphs below show both parametric (right column) and non-parametric analyses (left column) of the Hotelling’s T^2^ test using functions from open-source spm1d package ([www.spm1d.org](http://www.spm1d.org)) for walking, sit-to-stand, lowering and lifting. Each row in the graphs represents, in this order: Hip, Knee, Ankle, Upper Thoracic, Lower Thoracic, Upper Lumbar and Lower Lumbar.

**Walk:**

**
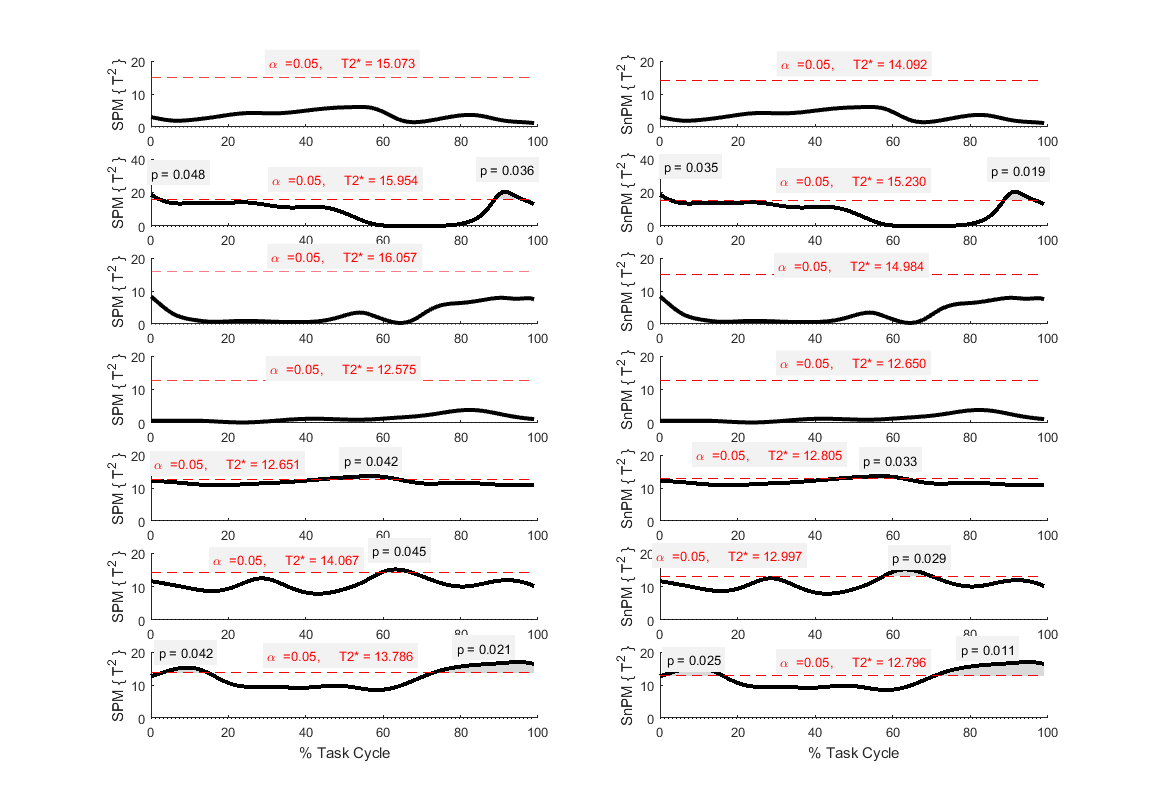
**

**Sit-to-Stand:**

**
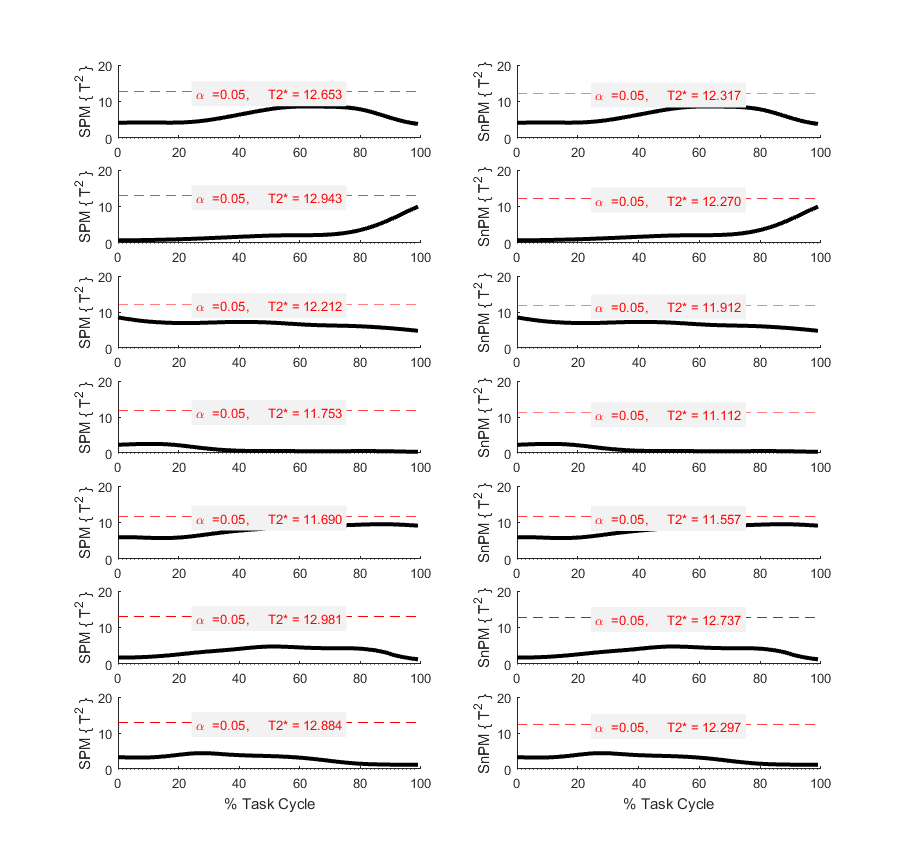
**

**Lowering:**

**
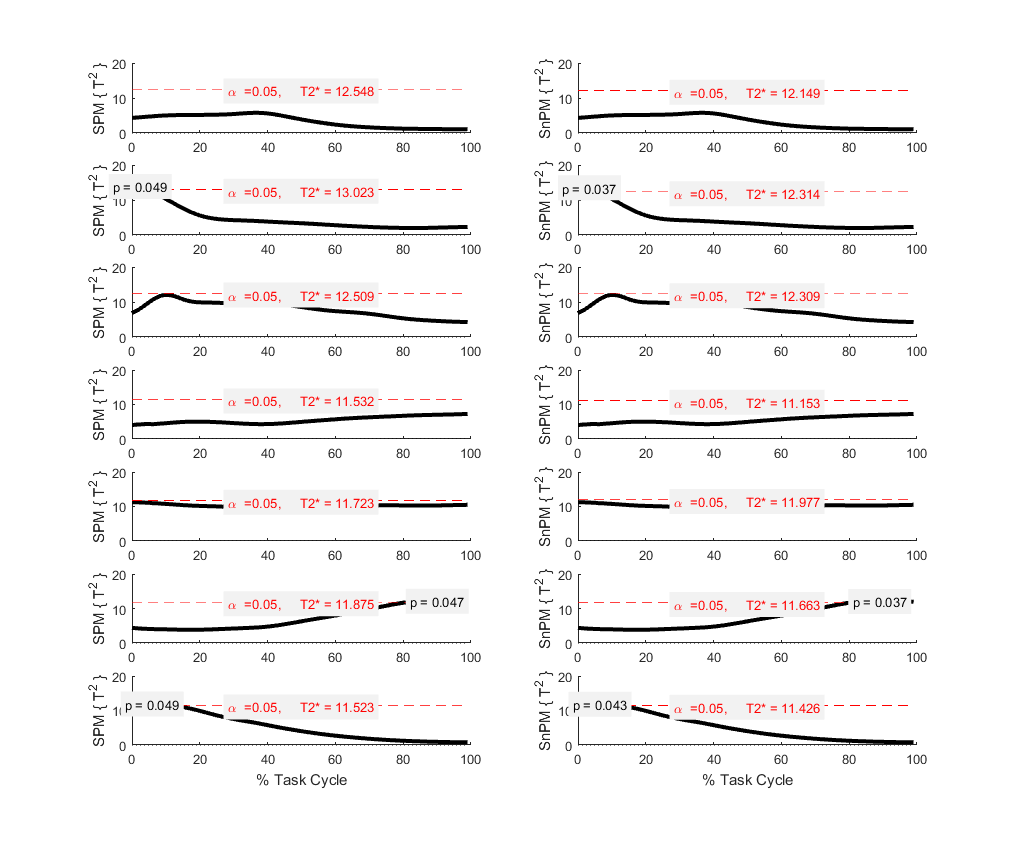
**

**Picking:**

**
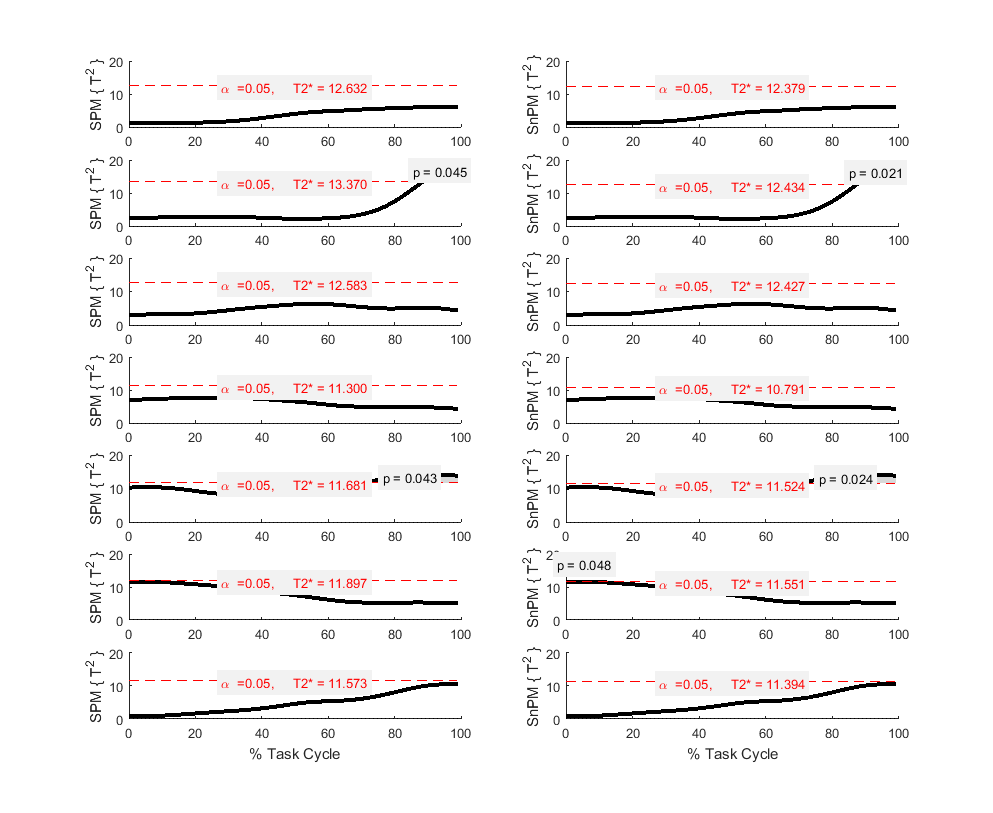
**
